# Phylogenetic Core Groups: a promising concept in search of a consistent methodological framework: Comment to ``A conceptual framework for the phylogenetically-constrained assembly of microbial communities''

**DOI:** 10.1186/s40168-021-01023-y

**Published:** 2021-03-25

**Authors:** Alberto Pascual-García

**Affiliations:** Institute of Integrative Biology, ETH-Zürich, Zürich, Switzerland

## Abstract

In this comment, we analyse the conceptual framework proposed by Aguirre de Cárcer (Microbiome 7:142, 2019), introducing the novel concept of Phylogenetic Core Groups (PCGs). This notion aims to complement the traditional classification in operational taxonomic units (OTUs), widely used in microbial ecology, to provide a more intrinsic taxonomical classification which avoids the use of pre-determined thresholds. However, to introduce this concept, the author frames his proposal in a wider theoretical framework based on a conceptualization of selection that we argue is a tautology. This blurs the subsequent formulation of an assembly principle for microbial communities, favouring that some contradictory examples introduced to support the framework appear aligned in their conclusions. And more importantly, under this framework and its derived methodology, it is not possible to infer PCGs from data in a consistent way. We reanalyse the proposal to identify its logical and methodological flaws and, through the analysis of synthetic scenarios, we propose a number of methodological refinements to contribute towards the determination of PCGs in a consistent way. We hope our analysis will promote the exploration of PCGs as a potentially valuable tool, helping to bridge the gap between environmental conditions and community composition in microbial ecology.

Video Abstract

**Supplementary Information:**

The online version contains supplementary material available at (10.1186/s40168-021-01023-y).

## Background

“The mission of community ecology, as of any scientific endeavor, is to detect the patterns of natural systems, to explain the causal processes that underlie them, and to generalize these explanations as far as possible.” John Wiens.

Roger Lewin started with this quote his article “Santa Rosalia was a goat”, reviewing twenty years of controversy in community ecology [1]. Heading the two confronting sides were, on the one hand, Jared Diamond, who, in 1975, proposed seven rules summarizing a number of observations which attempted to explain the assemblage of birds’ species in islands on the basis of competitive processes [2]. On the other hand, Daniel Simberloff and Edward F. Connor criticized Diamond’s work. Their claim was that every assembly rule was “either tautological, trivial, or a pattern expected were species distributed at random” [3]. They reasoned—following a Popperian prospect—that to demonstrate the role of competition requires rejecting a null hypothesis which considers that the observed patterns are not the result of purely stochastic processes [3]. Their work prompted the development of null models [4] and the proposal of the Neutral Theory of Biodiversity by Stephen Hubbell [5], both widely adopted approximations in nowadays community ecology.

Thanks to the development of new technologies such as next generation sequencing, we are in a privileged position to start deciphering the mechanisms underlying microbial communities’ assemblage, and novel conceptual frameworks, as the one presented by Aguirre de Cárcer in [6], are very welcome. However, some of the caveats that Simberloff and Connor considered in their analysis of Diamond’s work can be extrapolated to his work. In this comment, we identify some logical inconsistencies in the formulation of Aguirre de Cárcer’s framework, and we link them with the results he presents as verifications. Afterwards, we reanalyse the rationale of the formulation with synthetic examples, proposing a methodological pipeline to formulate different potential scenarios in which the framework may lie. By doing so, our hope is to improve the consistency of future formulations of microbial assembly principles.

### The proposed conceptualization of selection is a tautology

In the article ([6]), Aguirre de Cárcer first presents a number of observations that can be summarized as follows: microbial communities are phylogenetically clustered because certain traits are needed in any species in order to occupy a niche, and these traits are (to some extent) phylogenetically conserved. These observations led him to derive a microbial community assembly principle in the section “A new microbial community assembly principle”. Nevertheless, it is hard to say which is “the principle” because it is not explicitly enunciated as such. The author starts “conceptualiz(ing) selection as divisible into two niche categories: (i) niches whose occupancy requires specific phylogenetically conserved set of traits (from now on ‘phylo-niches’) and (ii) niches whose occupancy requires specific sets of traits not showing strong phylogenetic conservation (from now on ‘nonphylo-niches’)”. Following, we show that this formulation is a tautology. To simplify the explanation, we use variables to identify selection, *x*, and the two statements conceptualizing it will be described with the variables *a*= “statement (i)” and *b*= “statement (ii)”. Since the statements *a* and *b* are mutually exclusive (i.e. *b*=¬*a*, with ¬ being the operator NOT), this means that selection is simultaneously one thing and the contrary, *x*={*a*,¬*a*}. Therefore, selection (*x*) can escape from being a tautology if any of these possibilities are realistic alternative models: if there exist environments containing *only* phylo-niches (*x*={*a*}), if there exist environments containing *only* nonphylo-niches (*x*={¬*a*}), or if there are niches whose occupancy *does not* require specific sets of traits (*x*≠{*a*,¬*a*}). The mentioned possibilities are evaluated below.

To start with, the author does not justify why one environment should have both types of niches, and not only one. He highlights that, as a microbial ecosystem “can” present both types of niches, “each instance” of the same ecosystem type “should” present populations from both types of niches. Moreover, he does not explain what kind of conditions (biotic or abiotic) are expected to lead to phylo-niches or nonphylo-niches (which would be of a great interest). Therefore, we do not find arguments to substantiate that *x*={*a*}, or *x*={¬*a*} are valid alternatives. Secondly, we note that the author refers to phylogenetically conserved sets of traits (hereafter, phylogenetic signal, sensu Blomberg [7]) and not to phylogenetically related species. Therefore, the alternative model *x*≠{*a*,¬*a*} states that there exist niches occupied by species which do *not* share any minimum set of traits that is needed to occupy the niche (e.g. the traits found in a niche are random). This means that the proposed conceptualization of selection *x*={*a*,¬*a*} suffers from the same weakness as Diamond’s first rule (“If one considers all the combinations that can be formed from a group of related species, only certain ones of these combinations exist in nature” [3]): it only leaves space to build alternative hypotheses via completely random processes. Considering the strong evidence in the literature of the relationship between phylogenetic signal and phylogenetic relatedness and its role in adaptation [7], it seems unlikely to accept *x*≠{*a*,¬*a*}.

One still may argue that this conceptualization is not “the principle” but just a description of the niches that can be observed, which is needed to build the principle and to bring testable predictions. In this respect, the author continues presenting the following statements as predictions: (a) “any microbial ecosystem can present phylo-niches and nonphylo-niches; (b) for each phylo-niche there must be a discrete portion of the phylogeny (from now on a ‘phylogenetic core group’ or PCG) whose members share a phylogenetically conserved set of traits allowing the occupancy of their respective phylo-niche. (c) For each nonphylo-niche there must be a group of microbial populations sharing a set of traits not showing phylogenetic conservation allowing the occupancy of their respective nonphylo-niche.” It is easy to see that these are not predictions, because they are true by construction of the principle *x*={*a*,¬*a*} (note that the existence of PCGs is a direct consequence of the definition of phylo-niche), confirming that this conceptualization and its predictions constitute a logical trap.

### An imprecise formulation leads to the consideration of contradictory examples as verifications

At the end of the section above mentioned, we find a candidate-formulation of the principle: “Each instance of the same microbial ecosystem type should present populations from each PCG (occupying phylo-niches), and non-phylogenetic-core populations (occupying nonphylo-niches)”. As we anticipated, the reason why each “instance” “should” present both types of populations is not justified, although it is a testable prediction (both *x*={*a*}, and *x*={¬*a*} would reject the principle). The text then continues noting that, independently of the type of niche considered, species occupying a niche have similar traits and, hence, each group “should present a high degree of intra-group ecological coherence” (understood as similar life-strategies), and he postulates that the assembly within these niches “should be governed by neutral processes, and likely show intra-group competition.”

Considering these observations, we may define the principle by saying that microbial communities are assembled in niches through neutral processes (which may include competition, a possibility considered by the Unified Neutral Theory of Biodiversity [5]), with the species in these niches having or not phylogenetic signal, but for which it should be possible, in all cases, to identify relevant traits that are needed to occupy the niche. The section where “the principle” is presented finishes here and, at this point, it is difficult to reconcile this principle with statements such as (see abstract): “deterministic processes have a significant impact on microbial community assembly” without further clarifications.

For instance, our understanding of this formulation nicely fits the competitive lottery model [8], in which phylogenetic relatedness is not a requirement [9]. Surprisingly, despite of the fact that the mechanisms postulated in Ref. [9] are similar, they are presented by the author as a result that is inconsistent with the framework, due to the high phylogenetic overdispersion found (which may be, however, compatible with other scenarios we discuss below). This lack of consistency is possibly the reason behind the author’s proposal of a “second tier” of observations considering patchiness, biotic interactions and higher-scale sampling, to overcome these difficulties. In our view, a more exhaustive examination of the different scenarios is needed, combining phylogenetic-signal and the interplay between selection and stochastic processes and, perhaps more importantly, the proposal of a methodological pipeline independent from the number of samples or its depth. These are the challenges we address in the next sections.

### Neutral theory, the relation between traits and environmental conditions and “amendments” of the theory

Leaving aside the conceptual weaknesses of the formulation, Aguirre de Cárcer presents a potentially sound proposal under the notion of Phylogenetic Core Groups (PCGs). Given a set of 16S rRNA samples, he proposes to cluster 16S rRNA sequences as follows [6, 10]: OTUs are determined by hierarchically clustering the sequences at different similarity thresholds, and PCGs are clusters of sequences found in all samples at the maximum similarity starting from shallow levels, and proceeding towards deeper levels, after removing the sequences already clustered at higher sequence similarities. Identification of traits can be performed by analysing predicted metagenomes with a method like PiCRUST [11], also used by Aguirre de Cárcer in [10].

The idea departs from a simple assumption: if some microbes in a given sample are observed, they must have traits which encode the functions that are needed to occupy the environment from which they were sampled. However, it is unclear why determining PCGs will lead to a better understanding of which are the underlying environmental conditions or how to identify the relevant traits with them. For example, convergent evolution may lead to different traits being used by unrelated species to cope with a given environment. In addition, the procedure strongly depends on the number of samples considered, as we anticipated.

**Fig. 1:**
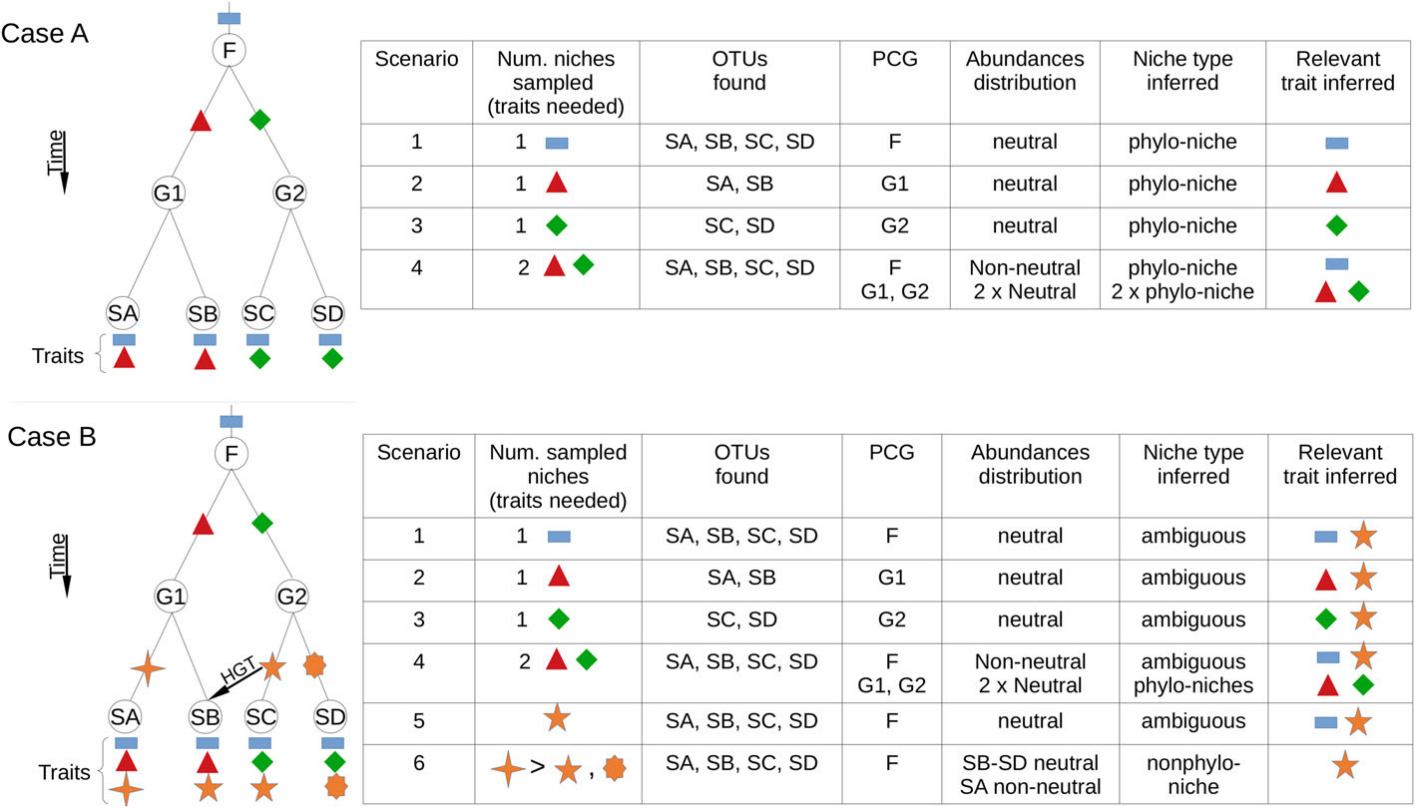
Analysis of synthetic scenarios. Two evolutionary histories (left column) of four species with traits, either appearing through duplication and divergent selection (case A), or also considering convergence and horizontal gene transfer (case B). The different scenarios take sampled niches (described by the traits which are needed to inhabit them) and, depending on the OTUs found and on the analysis of neutrality, the inferred PCGs and types of niches are shown. These scenarios are analysed in detail in the main text

To better understand these caveats, in Fig. 1, we present a synthetic example in which a microbial community, clustered^1^ at the family level “F”, diversifies into two genera, “G1” and “G2”, and, subsequently, into four species “SA-SD”. Traits are represented with different shapes, and their function encoded with a colour, and they are located in the evolutionary branch from which they emerge. To simplify the exercise, and without loss of generality, we assume that species are functionally equivalent if the relevant trait(s) needed to occupy a niche is (are) the same. This is a basic assumption for assemblies governed by neutral processes. The traits identified for each species are shown under the leaves of the tree.

Following the above conceptualization of selection, to occupy a niche requires a set of traits (conserved or not), and this map allows us to label a niche by using the required set of traits (second column in Fig. 1). Then, we perform a mental exercise in which we ask what conclusions can be drawn about the existence of the types of niches considered in the principle. And, to address this question, we take into consideration (i) if one or several niches were sampled; (ii) which species were found; (iii) their relatedness and their traits, and (iv) we further perform a test of neutrality, considering the species distributions.

The example is divided in two cases (A and B), each of which contains different scenarios (Fig. 1). Each scenario considers a set of samples, and the identification of operational taxonomic units and PCGs is performed using 16S rRNA sequencing and following the author’s prospect explained above. In case A (see Fig. 1), we identified two conserved traits for each species. Scenario 1 sampled all four species and, since all species are equivalent in this environment (only the rectangle-trait is relevant, and it is shared by all of them), we expect to find a positive test of neutrality, what immediately leads us to conclude that it is a phylo-niche: the rectangle is the relevant trait which is needed to survive, and the PCG is the whole family F. Scenarios 2 and 3 sample only two species each, and, again, we will expect a positive neutrality test for each of them. The absence of the other two species in each case, will lead us to conclude that the relevant traits are the triangle and the diamond, and the PCGs, the genus G1 and G2, respectively.

Scenario 4 samples two niches and, without further analysis, the search for a common PCG would lead us to conclude that the rectangle is again the relevant trait, with the PCG being the whole family F (first row). Moreover, it is clear that this conclusion depends on the number of samples considered, because subsets of samples within this scenario may lead to scenarios 2 or 3 if only two species are found in these subsets, a caveat acknowledged by the author in his proposal, but not solved in practice. A possibility to conclude which is the scenario, is to perform a neutrality test in which all the samples are considered to build a metacommunity (see e.g. [13]) and, since two niches were sampled, we expect a departure from neutrality, as it was pointed out in [13] (Fig. 1, scenario 4, first row). After finding a method to separate these two subsets of samples (see next section), it would be possible to infer one metacommunity for each subset and, eventually, to find they are compatible with independent neutral models (scenario 4, second row), providing strong indication that two niches were sampled. This scenario is compatible with the competitive lottery model discussed above [8].

In case B (Fig. 1), we explore the identification of nonphylo-niches, considering one trait (encoded in the different stars, all of them performing the yellow function) as the result of species having convergent traits (appearing independently in the different branches), except for species B, which acquires the gene through horizontal gene transfer (HGT) from the branch leading to species C. Following the same reasoning than in the examples studied in case A, we can observe that it is not possible to conclude if the niches are phylo-niches or nonphylo-niches for scenarios 1–3, the reason being that the traits evolutionarily conserved and the star-trait are present in all species. However, performing a clustering and the neutral tests previously suggested will allow us to, again, neglect the star-trait as the relevant one in scenario 4, identifying the two phylo-niches associated to the diamond and square traits.

Let us finally consider two additional scenarios, 5 and 6, in which the star-trait is required to occupy the niche. Scenario 5 suffers from the same ambiguities than scenarios 1–3, and only increasing the number of samples may allow us to resolve them. For instance, finding samples in which species from a different family appear (i.e. not having the rectangle-trait, but having the star-trait) will allow us to identify the scenario as a nonphylo-niche. We may find phylogenetic overdispersion in this situation, as in [9]. Scenario 6 considers that, among the star traits, the one of species A is more efficient in any respect than the one of the remainder species (indicated with a > symbol), bringing it a selective advantage. Under these circumstances, we could expect neutrality being globally rejected, because species A departs from what is expected from a neutral model. However, the remainder species B–D could be functionally equivalent, and a deeper look may lead to observe that are compatible with a neutral model after excluding species A.

Navigating the examples presented by Aguirre de Cárcer to support his principle, it is possible to observe that the lack of precision in its formulation facilitates the consideration of very different examples as a support for his theory. For instance, results as those presented in [13, 14] possibly belong to scenario 6, which is one of the most complicated examples to fit within the proposed classification of niches. Aguirre de Cárcer states that “Burns and co-workers [38] showed that (...) certain gut bacterial OTU abundances significantly deviated from predictions under a neutral model” and also that “Harris et al. [46] (...) found that significant departure from predictions under a neutral model appeared at different taxonomic levels”. These examples report a departure from neutrality, which is not straightforward to reconcile with his formulation. Moreover, other examples as those in [15] can be considered canonical examples of niche theory, hardly framed within a neutral framework. As an example, in a follow-up of [15], we showed that, in experiments in which communities were grown in synthetic marine particles made of a mix of two substrates, it is possible to predict the distribution of the most abundant species as a linear combination of their abundances in single-substrates experiments [16]. This predictability in the assembly is hardly explained within a neutral framework. In summary, the fact that the theory proposed makes a strong emphasis into phylogenetic relatedness (which points towards niche selection), and the postulate that neutral processes are relevant, demand a more precise formulation on how both perspectives are integrated, given that there are multiple possible scenarios and some of them will lead to ambiguous conclusions, as we illustrated.

There are two last important questions that remain unclear in the theory. Firstly, the role of cooperative interactions and facilitation, whose important role is increasingly recognized (see e.g. [17]), and that seem to be introduced in the theory as some sort of “ammendments”. The difficulty to integrate these processes comes from the fact that it is unclear how, for example, species coexisting in the same niche via their reciprocal benefit are phylogenetically related, which environmental conditions favour their coexistence, or which are the expectations from neutral models [18]. Secondly, another important question is which are the evolutionary scenarios foreseen by the theory for speciation to occur, which is completely left at the discretion of the reader. Nevertheless, criticisms claiming that evolutionary and ecological mechanisms have been decoupled in the literature of macroscopic organisms [19] are even more pertinent in the microbial world, in which the evolutionary time-scales are much shorter. In particular, the existence of phylo-niches and nonphylo-niches should be framed within an evolutionary model to explain its formation.

### PCGs: a promising concept in search of a consistent methodology

From the previous analysis, we identify two key questions which deserve a closer look. To start with, a point we already highlighted, namely that this strategy strongly depends on the number of samples and their depth. Since this question is technical in nature, we will attempt to address it below. The second question would be what can be gained from this methodology that could not be addressed analysing directly all traits present in the set of samples under analysis.

A possible answer for the second question comes from the low taxonomic resolution that can be achieved sequencing fragments of the 16S rRNA gene, which cannot reliably resolve ranks under the genus level [12], in particular when the V4 region is targeted [20]. Since ecological coherence of taxa above the species level have revealed biogeographic patterns [21], estimating PCGs may be considered a more robust method to identify ecologically meaningful OTUs, further simplifying a subsequent search in the space of traits.

In addition, to the best of our knowledge, it still remains largely unknown for natural microbial communities how the degree of phylogenetic conservation expected relates to the dominance of homogeneous or heterogeneous selection or neutral processes. Therefore, another potentially interesting justification for searching PCGs is if their structure (i.e. the number and similarity thresholds of the OTUs they host) is informative of the underlying ecological and evolutionary processes.

**Fig. 2:**
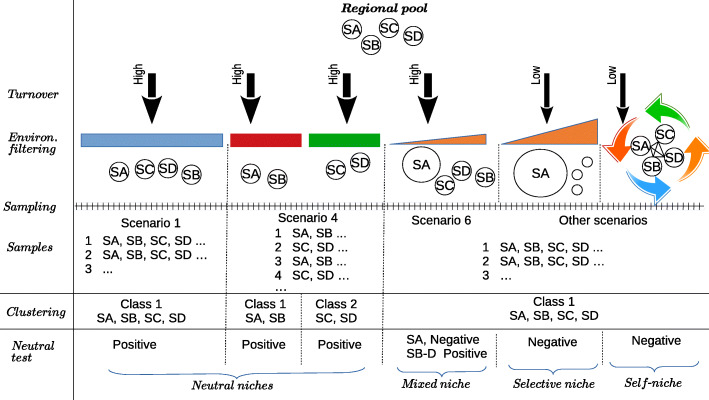
Summary of the proposed methodology. Starting from a regional pool of species (top), the immigration rate (turnover high or low) and the environmental filtering determine the species that can be observed. The environment may be homogeneous (coloured rectangles), heterogeneous (triangles), or may be shaped by continuous transformations due to strong feedbacks with the microbial activity (cyclic arrows). Sampling these environments, classifying the samples into classes, and performing neutrality tests will lead to a classification of environments into different types of niches

In the following, we attempt to produce a pipeline to incorporate to Aguirre de Cárcer’s proposal a more consistent methodology that is less sensible to the number of samples in the dataset. In addition, we propose a number of tests, preceding the determination of PCGs, that would help establish bridges between different assembly ecological processes and the phylogenetic signal. We illustrate the proposal in Fig. 2, in which we reproduce some of the scenarios discussed in Fig. 1.

Our proposal starts observing that the large number of different taxa in most natural samples makes unlikely to systematically find similar communities by chance. As a consequence, the determination of clusters of communities (hereafter “community classes”) sharing a significantly higher *β*−diversity similarity within classes than between classes is an appropriate starting point to infer the processes driving this similarity (see e.g [22, 23]). The *β*−diversity similarity should be computed using exact sequence variants (ESVs), i.e. sequences with single-nucleotide differences over the sequenced gene region [24], to avoid biases induced from a phylogenetic clustering [25]. The rationale is that the existence of classes can be indicative of environmental filters selecting for different communities’ compositions and also performing differentiated community-level functions [26].

Note that this strategy is valid only under the assumption that the study does not aim to disentangle the existence of sampled niches along a continuous environmental gradient, in which case, more complex *β*−diversity analyses should be considered to understand the effects of these gradients (reviewed in [27]). Another reason for determining classes is that the detection of phylogenetic signal depends on the fraction of the phylogenetic tree sampled [28], and, hence, an objective method is needed in order to determine the number of samples to be considered in the search of PCGs. Although the number of classes may increase (decrease) if a larger (smaller) number of samples are considered, there will always be a core of samples representative of each class. This fact will make the determination of PCGs within each class a more robust choice than directly using the whole set of samples.

The next step considers the realization of a neutrality test, which is affordable for large sets of samples with nowadays methods; see e.g. [13]. If classes exists, one metacommunity (in which speciation occurs) should be reconstructed for every class to investigate if species within each class are functionally equivalent (since species belonging to different classes are not—following the previous reasoning). If classes cannot be determined, the test should be instead applied to the whole set of samples.

Taking together the results from the test of neutrality and from the search of classes, it is possible to interpret them as follows. If classes cannot be determined and the test of neutrality is positive, there is a single environmental filter (large blue rectangle in Fig. 2, scenario 1) with possibly a high species turnover (large arrow in Fig. 2). If classes can be determined and the test is positive (Figs. 1 and 2, scenario 4), we are likely sampling several homogeneous niches (large red and green rectangles). The traits found will depend on the selective intensity of the environment. For example, if we assume that the areas sampled in scenarios 1 and 4 are similar, it is likely that niches in scenario 4 are spatially narrower (and hence, that there will be stronger competition), being the specific traits needed to occupy these niches positioned closer to the leaves of the phylogenetic tree than in scenario 1 (rectangle vs. diamond and triangle traits in Fig. 1). We may call niches described by scenarios 1 and 4 “neutral phylo-niches”, if the phylogenetic signal goes hand in hand with phylogenetic relatedness, or “neutral nonphylo-niches”, if phylogenetic overdispersion is found.

If the neutral test fails, independently of the existence of classes, we could investigate if departure from neutrality comes from subsets of species (scenario 6) or from the community as a whole (“other scenarios” in Fig. 2). If only subsets of species depart from neutrality, the environment is not uniform in the selective pressure it exerts (environmental gradient illustrated as a triangle), meaning that species having a more specialized adaptation have a selective advantage, and these species appear to be more abundant than expected under a neutral scenario. In addition, if turnover is high (as it is the case in the example of gut’s microbiome discussed above), the remainder species’ distributions could be described with a neutral model. These environments may also be compatible with both phylo-niches and nonphylo-niches, and then, the dichotomy should be elucidated with a more detailed phylogenetic analysis. We call this scenario a mixed (neutral and selective) (non)phylo-niche.

An additional test that can help to disentangle the dichotomy between neutral processes and selection comes from the computation of the Nearest Taxon Index [29]. The index computes the *Z*-score of the mean phylogenetic distance of the taxa present in the community with respect to its random expectation. A significantly positive value is expected for an heterogeneous selective environment and significantly negative for an homogeneous selective environment. For example, in environments where resources are refreshed after long time intervals, we expect to find a transition from homogeneous (fresh resources) to heterogeneous (degraded resources), see e.g. [16]. An open question would be if, e.g. a homogeneous selective environment leads to phylo-niches, whereas non-phyloniches would be expected for a heterogeneous selective environment.

Finally, if neutrality is rejected, we are dealing with scenarios in which strong environmental selection, ecological interactions, or both, are dominant (“other scenarios” in Fig. 2). If environmental selection is the primary selective force (steep triangle in Fig. 2), the acquisition of specialized traits required to survive under certain environmental conditions should be reflected in a remarkable phylogenetic signal. We call this scenario a selective phylo-niche. If ecological interactions are prevalent, in particular syntrophy, the microbial community may effectively shape its own niche (circle of arrows in Fig. 2). Under this scenario, phylogenetic relatedness may be deep, since species phylogenetically distinct may play complementary roles (see e.g. [30, 31]). Strikingly, although the lack of functional equivalence suggests that neutrality should be rejected, theoretical results found that the distribution of abundances of these communities could be compatible with a neutral model [32].

Following these scenarios in which we combine environmental selection, neutral processes, and ecological interactions, we could ask which are the distributions of PCGs expected for each of them. Simulations can be developed to create null expectations for each scenario, and, hence, the detection of PCGs could contribute to a better understanding of the natural conditions shaping natural communities. Although our proposal will make more clear the relation between subsets of samples, ecological processes, and environments, it remains unanswered how the relevant traits associated to each niche will be identified.

This question is typically assessed by looking for relevant environmental variables that correlate with certain traits. Alternatively, comparing the relative frequencies of traits between community classes has been proved a useful approximation to identify ecological processes [26]. In our opinion, it is unclear if the identification of PCGs brings a more direct pathway to identify the relevant traits than other approximations.

### Conclusion

In this comment, we analysed the conceptual proposal of Aguirre de Cárcer showing that the lack of precision in its formulation leads to a logical conundrum. Despite of this observation, we found an interesting avenue to follow which exploits the concept of PCGs to shift the attention from the traditional OTUs’ analysis to the identification of a more intrinsic level of organization.

We showed that there is no clear reason to expect that PCGs reflect any specific ecological or environmental processes and that their characterization must be independent of the number of samples considered and their depth. To address these caveats, we argued that the search for PCGs could be performed after characterizing community classes and once tests aiming to disentangle ecological and environmental processes (e.g. neutral tests, or the computation of the NTI metric) are performed on each class. Both the identification of classes and the tests of neutrality are robust against an increasing number and depth of samples, provided that these are sufficient (see, e.g. tests with different sampling depths in [13]).

It remains, however, unclear, which benefit may bring their characterization. Working with ESVs represents a better standard to compare biodiversity patterns across different environments [33] than any OTU-based approximation (including PCGs). From the point of view of the determination of traits, it should be tested if there is any gain determining PCGs instead of, e.g. directly inferring traits for each exact sequence variant and, then, looking at which functions are present in each class, and their relative differences between classes. This question is particularly relevant after observing that ESVs that would be clustered together into an OTU (97% sequence identity) can have different dynamics [34]. Although it remains unclear if different dynamics reflect different ecological strategies or are simply explained by processes such as priority effects, two bacteria with none or few substitutions in the 16S rRNA gene may have different genomes and hence different life histories. Therefore, the interest of approximations such as the determination of PCGs rely on the assumption that ecological coherence is observed at high taxonomy ranks [21]. If the development of more accurate sequencing and computational methods reveal that this assumption is challenged, it will possibly be needed to combine 16S rRNA sequencing with other sequencing methods, such as metagenomics or whole-genome sequencing, to have an accurate description of biogeographic and community-level functional patterns.

A final possible application would come if it is shown that PCGs structure is determined by the underlying environmental and ecological processes structuring communities. This would allow us to characterize them directly without running into the different steps of the pipeline proposed here. To address this question, possible different scenarios could be simulated to determine null expectations, and if clear patterns appear for PCGs, it would be a valuable tool to link selection with the search of traits, a much needed approximation to investigate community-level function. Until these questions are addressed, PCGs will be an appealing concept, opening more questions than the answers it provides.

## Data Availability

Not applicable Declarations

## Footnotes

1 For instance, as it is customary for 16S rRNA amplicon sequencing sampling experiments in which different taxonomic levels are determined on the basis of the gene similarity at different thresholds, although we acknowledge the current limitations of this approach to resolve the taxonomy below the genus level [12]
